# A well-being support program for patients with severe mental illness: a service evaluation

**DOI:** 10.1186/1471-244X-11-46

**Published:** 2011-03-21

**Authors:** Donna Eldridge, Nicky Dawber, Richard Gray

**Affiliations:** 1 Kent and Medway NHS and Social Care Partnership Trust, UK; 2 Faculty of Health, University of East Anglia, Norwich, NR4 7TJ, UK

## Abstract

**Background:**

The risk of cardiovascular disease is increased in patients with severe mental illness (SMI) dramatically reducing life expectancy.

**Method:**

A real world pragmatic service evaluation of a Well-Being Support Program (WSP) was conducted. This was a four-session package delivered over a one-year period by mental health practitioners that had received additional training in providing physical health assessment and intervention. Patients' physical health was screened and appropriate one-to-one and group intervention was offered.

**Results:**

212 mental health practitioners were trained in the WSP and 782 patients were enrolled on the program. The majority of our sample was overweight or obese; 66% had a Body Mass Index (BMI) >25. Lifestyle risk factors for cardiovascular disease (CVD) were common and the patients had low self esteem. The average number of formally recorded well-being sessions attended was 2.10. Just under a quarter of those patients enrolled in the program completed. The only cardiovascular risk factor that significantly altered in patients that completed the program was BMI. The qualitative feedback about the program was largely positive.

**Conclusions:**

The need to intervene to enhance the physical health of people with SMI is beyond doubt. Maintaining patient engagement in a physical health improvement program is challenging. Regular comprehensive physical health monitoring is necessary to establish the benefit of intervention and increase life expectancy and well-being in this population.

## Background

Life expectancy for people with severe mental illness (SMI), such as schizophrenia is reduced by at least 20% compared with the general population [1,2]. The major cause of death in this population is cardiovascular disease (CVD) [3,4]. Just having a SMI may increase the risk of CVD but this inherent vulnerability is compounded by lifestyle factors that include a high fat and high calorie diet, lack of exercise, smoking and substance use [5-7]. Weight gain and other side effects of antipsychotic medications used to treat SMI further add to the cardiovascular burden [8]. Recognizing and managing risk factors for CVD and other physical co-morbidities is an important unmet need in the SMI population [9]. Despite frequent contact with primary care services, physical health is rarely monitored [10-12] an observation that may be explained by diagnostic overshadowing (where presenting symptoms are put down to the mental illness). SMI patients are generally in regular contact with community mental health workers who may be better placed to both monitor and promote physical wellbeing [13]. There is evidence that that mental health workers consider the physical health of their patients an important part of their role [14].

A Cochrane review [15] of physical health monitoring for patients with SMI concluded that there was no evidence from RCTs to support current practice. Physical health screening programs for SMI patients in secondary care have been described by Millar [16] and Shuel et al [17]. In both, high rates of obesity and other CVD risk factors were observed and a high degree of patient appreciation for the programs was reported. Several exploratory evaluations of physical health interventions designed or adapted to be delivered in secondary care have been described [18-21]. For example, Ball et al [18] compared a "Weight Watchers" program in 11 schizophrenia and 11 matched control patients but did not observe significant weight loss as a result of the intervention. A service evaluation by Pendlebury et al [19] reported a mean reduction in weight of just over 6 kg in a sample of 93 SMI patients attending a mean of 42 behavioral weight management clinic sessions.

The Wellbeing Support Program (WSP) described by Ohlsen et al [20] is perhaps the most important described in the literature (because of the number of service providers in the UK that have gone on to adopt the program). The WSP is a nurse led screening service that aimed to:

• Identify physical health problems,

• Promote treatment adherence,

• Encourage positive lifestyle change,

• Strengthen links between primary and secondary care

• Provide support and advice to carers

• Direct patients to appropriate primary and secondary care services.

The service was facilitated by a team of nurse advisors who provided Well-Being support as an adjunct to routine care. Program development, support for the nurse advisors and subsequent evaluation was supported by the pharmaceutical company Eli-Lilly who manufacture an antipsychotic medciation used to treat schizophrenia.

Delivered over a two year period the WSP is a five-step program with a minimum of six face-to-face sessions with a nurse wellbeing worker:

• Step 1: SMI patients are invited to participate and enrolled on the WSP register

• Step 2: the first face-to-face well-being session where physical health (blood pressure, pulse, weight and height), lifestyle factors (diet, physical activity, smoking status) are measured.

• Step 3: results (from session 1) are fed back to patients at a second face-to-face session. Blood tests (random blood glucose, thyroid function, liver function, serum prolactin, lipid screen) are performed during this meeting

• Step 4: patients are referred by the practitioner to one or more of the following: a weight management or physical activity group, primary care or specialist doctor for additional physical health care, or medication review by prescribing clinician

◦ Weight management groups are held weekly; patients are weighed and there is an opportunity to access support information and advice.

◦ Physical activity groups were also held weekly. Activities including, bowling, badminton, walking and swimming were organized by the nurse.

• Step 5: follow-up face-to-face session(s) to evaluate the program and complete follow-up measures (as in step 1 and 2).

Patient progression through and adherence to the key elements of the five stages of the program was carefully monitored by the nurse advisors managers. Treatment and training manuals and fidelity measures for the WSP have not (to date) been published.

Smith *et al. *[21] addressed the question of whether the Well-Being Support Program (WSP) provided in a secondary care setting was effective in modifying lifestyle factors such as diet, lack of exercise and cigarette smoking. Outcomes of the service were positive and encouraging. Of the 966 patients enrolled, 80% completed the program, and there were significant improvements in physical activity, smoking and diet [13,21]. It might be argued that rather than advocating the rolling out the program Well-Being Support should be subject to a randomized controlled trial. Whilst there is considerable merit to this argument many service providers were convinced that the evidence from the service evaluation was sufficient to warrent adoption. Although we know anecdotally that many secondary mental health services in the UK have adopted the WSP actual numbers have not been published. As Tosh et al [15] note there has been considerable financial investment by service provider in enhancing the physical health of SMI patients with minimal evidence establishing effectiveness or cost effectiveness of any intervention or programme.

The Kent and Medway National Health Service (NHS) and Social Care Partnership Trust, is a typical secondary mental health service provider to a population of 1.6 million in the South East of England. At any given point there are approximately 25,000 open cases and around 4,000 members of staff employed in the Trust. Prior to implementing the WSP, there was minimal physical health care provided to patients that used the service. There was considerable debate within clinical teams, management groups and the executive team about how to address the physical health needs of patients using Trust services. Despite the lack of evidence from clinical trials practitioners that had attended presentations about the WSP were keen advocates of the program. Support from clinicians and subsequent endorsement by the Department of Health convinced the Trust to implement the program.

For pragmatic (not scientific) reasons a number of adaptations to the WSP were made by the Trust prior to implementation:

• Mental health practitioners in routine practice (not Nurse advisors) would deliver the program

• Patients would be offered four and not six face to face Well-Being sessions

• The length of the program would be reduced from two to one year

• MHPs receive three days training to deliver the program by a Nurse Advisor who had worked on the Smith et al [21] project

• MHPs would deliver the program directly to patients on their caseload

• Adherence to each of the five steps of the program would not be monitored

Adoption and implementation of the WSP represented a considerable financial investment by the Trust. We argue that it is important to determine if, when rolled out in a real world service, patients engage and benefit from the program.

### Primary aim

The primary aim of this investigation was to determine the proportion of patients that completed the WSP.

### Secondary aims

In addition, the project reviewed the effect of the WSP on a range of cardiovascular risk factors:

• BMI

• Hypertension

• Smoking status

• Alcohol use

• Substance use

• Physical activity

• Diet

• Self-esteem

### Tertiary aim

Additionally we conducted as series of in depth fact-to-face interviews with six WSP practitioners to help us better understand the strengths and weaknesses of the program and consider how it might be developed in the future.

## Methods

### Training of WSP practitioners

A series of WSP training courses for mental health practitioners were undertaken starting in September 2006 with an initial cohort of 50 (this was our initial recruitment target). Sending out flyers, contacting team leaders and word of mouth were the main methods used to recruit practitioners. Training lasted for three days and focused on equipping practitioners with the necessary physical health competencies to facilitate the program. A nurse advisor that had previously run the well-being clinical service led the training.

Practitioners only delivered the program to patients on their caseload, limiting the number of patients that could access the program. To reach more patients more practitioners needed to be trained. We therefore established a rolling program of training that any practitioner could apply to attend on a first come first served basis. By the end of 2008, 212 mental health workers were trained. The well-being support service was established in September 2006, and by September 2008, 754 patients were enrolled onto the program representing around 3% of the patients that use our services. Each worker recruited an average of 4 patients. Around 1 in 4 of the patients on an average practitioners' caseload. All patients that were enrolled in the WSP consented to participate and for data being used for the purposes of this service evaluation. Ethical approval was not required for this service evaluation.

Practitioners were responsible for recruiting patients from their caseload that had a severe mental illness diagnosis (schizophrenia or bipolar disorder) They were also responsible for registering patients and entering data onto a computerized Excel database concerning their progress through the WSP. Additionally, in order to develop a more in depth understanding of how practice was changed, six mental health practitioners were interviewed concerning the delivery of the program. Each interview was anonymous and consent was gained separately for this part of the study. Practitioners were asked for feedback about their experience of the WSP with open-ended questions which included: what they thought about the program, if they felt it had had any effect on practice and if they believed the program had any effect on patients' physical wellbeing. Interviews were conducted on a one-to-one basis in a private room and answers were recorded verbatim on an anonymous data collection form for later collation and analysis. Interviews took 10-20 minutes.

### Analysis

We considered improvement to be any categorical change (e.g. obese to overweight, smoker to non-smoker, low self-esteem to low/medium self esteem) in any of the cardiovascular outcomes that we measured. The researchers used a mixture of descriptive, chi-square tests for categorical variables and t-tests for continuous variables. Data were originally entered onto an Excel database and then transposed into PASW (SPSS) v17 for analysis. Practitioner interviews were subject to thematic analysis by two of the authors [DE/RG] to identify major themes.

## Results

We did not set a target for the number of patients we sought to enroll in the program. A total of 754 patients were registered on the program (Step 1) and attended a formal Well-Being session (Step 2). Step 3 (feedback on session 1 and blood tests) was attended by 297 (33%) patients, step 4 (referral) by 370 (49%) and the final one year follow up session by 159 (21%). It is note worthy that step 2 was less well attended than session 3, perhaps indicating reticence among patients to have bloods drawn. In total patients attended 1580 formal well-being consultations, a mean of 2.10 each, which equates to half of the program. We did not record information on how many weight management and exercise groups patients attended. Most participants were male, white and in their late 40 s (Table 1). The majority of patients were prescribed psychotropic medication, with antipsychotics being the most commonly prescribed (Table 2). On average, patients were prescribed 1.6 psychotropic medicines each. Feedback from participants suggested that there was considerable *ad hoc *intervention that was not captured by our data collection mechanism; mainly this consisted of attendance at weight management and exercise groups. A minority of enrolled patients completed the program (n = 159) representing a drop-out rate of 79% over one year. Although the prescribed duration of the program was one year, on average completion assessments were undertaken nine months (standard deviation = 6.85) after enrolment. Patients that completed the program appeared to recognize the need to address their physical well-being; being more likely to be overweight or obese and have an unhealthy diet.

**Table 1:** Baseline demographics

	n%
Gender (% male)	434 (55.5)
Ethnicity	
White	733 (94)
Black	13 (2)
Asian	20 (3)
Other	16 (2)
Age (mean s.d.)	46.24 (12.4)

s.d., standard deviation

**Table 2:** Prescribed psychotropic medication (n = 794)

Name/type of medicine	n (%)
*Antipsychotics*	
Risperidone	97 (12)
Olanzapine	115 (14)
Quetiapine	56 (7)
Amisulpride	44 (6)
Aripirazole	29 (4)
Clozapine	83 (10)
Typical depot (Piportal, zuclopenthixol)	107 (13)
Oral typical (sulpiride)	83 (10)

*Antidepressants*	
Antidepressant (SSRIs/SNRIs)	246 (31)
Antidepressant (TCAs/MAOIs)	30 (4)

*Medicines used for prophylaxis of bipolar illness*	
Sodium valproate, lithium, carbamazepine	151 (19)

*Other psychotropic medicines*	
Benzodiazepine	71 (9)
Anticholinergic	74 (9)

Mean number of medicines per patient	1.6

Total number of medicines prescribed	1186

SSRI, selective serotonin reuptake inhibitor; SNRI, serotonin-norepinephrine reuptake inhibitor; TCAs, tricyclic antidepressant; MAOI, monoamine oxidase inhibitor.

### Outcomes of the WSP

Rates of cardiovascular risk factors at baseline (for the group as a whole and for completers) and at the final (one year) consultation are shown in Table 3. Results for laboratory tests completed on approximately one-quarter of patients are shown in Table 4. Two-thirds of patients that participated in the program were overweight or obese, over a third had hypertension and a half (of those tested) had abnormal low-density lipoprotein (LDL) cholesterol. Almost half of the patients in our sample had an average/poor quality diet. A similar proportion did not take regular activity, smoked and drank alcohol. Of those patients who reported that they drank alcohol, the estimated average number of units drunk in a week was 46; considerably more than the recommended 21 units per week [22]. A modest number of patients reported that they used illicit substances; however, it is likely that this is an underestimate of the true levels of substance use, potentially because of patient concerns in answering the question honestly. Baseline laboratory results were only recorded for a minority of patients. Fasting, and if not possible non-fasting, blood samples were used in the study and normal ranges were defined by the laboratory that performed the testing. A modest but important number of patients tested had abnormal blood glucose (21%), triglycerides (32%) and prolactin (17%).

**Table 3:** Cardiovascular risk factors at program and completion

	Total (program entry = 754; completion n = 159)			Differences (df) (completers only)
		
	Program entry (n = 754,100%)	Program entry (completers n = 159, 20%)	Program completion (n = 159, 20%)	
BMI				
<24.9 (normal)	281 (37%)	41 (28%)	33 (26%)	χ^2 ^= 126.01; df = 4; p < 0.01
25-29.9 (overweight)	303 (40%)	29 (20%)	35 (28%)	
>30 (obese)	198 (26%)	76 (52%)	59 (47%)	
Hypertension				
Normal BP (<140/85)	494 (66%)	103 (70%)	88 (69%)	χ^2 ^= 15.01; df = 1; p = <0.01
Hypertension (>140/85)	260 (34%)	27 (30%)	39 (31%)	
Cigarette smoking				
Smoker	315 (48%)	65 (41%)	71 (45%)	ns
Non-smoker	331 (51%)	94 (59%)	88 (55%)	
Alcohol				
Alcohol	143 (22%)	22 (14%)	35 (22%)	ns
No-alcohol	503 (78%)	137 (86%)	124 (78%)	
Substance use				
Cannabis use	69 (11%)	5 (3%)	6 (4%)	ns
Other reported substance use (e.g. cocaine, ecstasy, heroin, amphetamine)	38 (6%)	8 (5%)	7 (4%)	ns
Activity				
No regular activity	284 (44%)	72 (45%)	62 (39%)	ns
Diet				
Unhealthy diet	104 (16%)	100 (63%)	88 (55%)	ns
Average diet	182 (28%)	35 (22%)	43 (27%)	
Healthy diet	360 (56%)	24 (15%)	28 (18%)	
Self-esteem				
Low self-esteem	330 (51%)	93 (59%)	82 (52%)	ns
Low/medium self-esteem	104 (16%)	30 (19%)	30 (19%)	
Medium self-esteem	181 (28%)	32 (20%)	42 (26%)	
Medium/high self-esteem	22 (3%)	4 (3%)	3 (2%)	
High self-esteem	9 (1%)	0 (0%)	2 (1%)	

BMI, body mass index; χ^2^, chi squared test; ns, not significant.

**Table 4:** Abnormal blood results at baseline

Baseline	n (%) abnormal*	Mean (s.d.)	Range
Random glucose (normal 4-7 mmol/l), n = 138	26 (19%)	5.57 (2.20)	3.10-20.30
Fasting glucose (normal 3.6-6.0 mmol/l), n = 39	8 (21%)	5.45 (1.60)	3.30-11.10
HbA_1c_, n = 29	0 (0%)	6.02 (1.43)	4.60-12.80
Triglyceride (reference range <2.0 mmol/l), n = 159	52 (32%)	2.31 (1.63)	0.50-10.50
HDL cholesterol (reference ranges male 0.9 to 1.4; female 1.2 to 1.7 mmol/l), n = 178	52 (29%)	1.34 (6.5)	0.70-5.40
LDL cholesterol (reference >4.0 mmol/l), n = 128	67 (52%)	5.29 (1.27)	1.80-9.00
Aspartate Aminotransferase (AST-reference ranges, male 8-40 IU/l; female 6-34), n = 7	0 (0%)	20.57 (4.79)	13.00-26.00
ALT (reference ranges male 10-50; female 5-38 IU/l), n = 180	16 (9%)	27.91 (18.78)	6.00-124.00
Total bilirubin (reference range 3-17*μ*mol/l), n = 167	3 (2%)	9.27 (3.44)	2.00-27.00
Albumin (reference range 35 to 50 g/l), n = 189	0 (0%)	42.43 (2.86)	36.00-50.00
Prolactin (mU/l; reference range 80-400 males; 90-520 females reproductive years; 80-280 females post menopausal), n = 129	22 (17%)	454.26 (542.62)	1.58-2862.00
Free T4 (reference range 9-25 pmol/l), n = 159	1 (1%)	14.37 (2.57)	5.00-25.00

*of those patients tested

ALT, alanine transaminase; HbA_1c_, hemoglobin A_1c_; HDL, high density lipoprotein; LDL, low density lipoprotein; s.d., standard deviation; TSH, thyroid stimulating hormone.

The researchers found significant correlations between body mass index (BMI) and blood pressure (BP) (correlation coefficient r = 0.22, p < 0.01); BMI and smoking, cannabis use and other illicit drug use (r = 0.14, p = 0.01; r = 0.12, p < 0.01; r = 0.11, p < 0.01; respectively); diet and exercise (r = 0.118, p < 0.01); and self esteem and exercise (r = 0.33, p < 0.01). There was a positive correlation between cannabis and use of other illicit substances (r = 0.34, p < 0.01) but a negative correlation between smoking cigarettes and cannabis (r = -0.26, p < 0.01) and other illicit substances (r = -0.15, p < 0.01).

Most patients were referred to the lifestyle (n = 621, 82%) and/or physical activity groups (n = 541, 72%), but the number of activity groups patients attended was not recorded. The other major intervention used by practitioners running the WSP was referral to a professional colleague. This included psychiatrists (or nurse prescribers) for medication review, and primary care colleagues for physical intervention or further investigations. On average, the final face-to-face session in the program was nine months (s.d. = 6.84) after patients were enrolled on the one-year program. Just under a quarter of the patients enrolled (n = 159, 21%) completed the program. In those who completed the program, researchers observed a significant categorical change in BMI in 23 out of 159 patients. Categorical improvement in BMI (e.g. overweight to normal) was observed in 15 patients (12%) completing the program. A categorical worsening (e.g. overweight to obese) in BMI was seen in eight patients (7%). Mean change in BMI was not statistically significant with a mean BMI at baseline of 30.05 (s.d. = 7.80) and at the end of the program of 29.86 (s.d. = 7.42), a change of 0.38. The only other significant association observed was in hypertension. A categorical improvement in BP (from hypertensive to normal) was observed in 17 patients (14%) and a worsening (from normal to hypertensive) in 18 (15%). The program appeared to have no effect on the proportion of patients that smoked, drank alcohol, used illicit substances, took exercise, the quality of diet or their self esteem (Table 2).

### Qualitative comments about WSP

Six WSP practitioners were interviewed to elicit their feedback. Four major themes were identified: making a difference, feedback from patients, working holistically to promote recovery and modification of the program.

### Making a difference

All of the practitioners who were interviewed talked about the need for physical health intervention for patients with SMI and what a positive difference the WSP had made to their practice.

"...it has flagged up a massive deficit within our clients with some having not had physical interventions for a number years. A lady I assessed hadn't had a smear for 20 years!" (Practitioner 3).

"...I have welcomed the program as it recognizes the need to provide health promotion activity to a client group where this can be overlooked, falling between services of secondary and primary care."

Participants also described how initial pessimism has transformed into positive clinical practice.

"Initially I was concerned that health and well-being would be training I had gone on early on in my post and would fall by the wayside or I would not be able to implement, particularly in this setting. However, fellow staff that went on the training and the link meetings have meant this has not been the case, which I am pleased about as running the group and working with others getting the project off the ground has been really enjoyable." (Practitioner 2)

There was also evidence that real changes have occurred in practice as a result of WSP.

"In fact we are cooking a healthy fry-up this week! (i.e. grilling not frying, including lots of vegetables and fresh fruit)..." (Practitioner 2)

It does not appear that practitioners that have done the WSP see themselves as experts in physical health. There is evidence that when appropriate, they make referrals to appropriate professional colleagues.

"...high blood sugar, hypertension, obesity, polypharmacy and sexual health problems all of which I have been able to refer to appropriate services". (Practitioner 3)

"...I have discovered a number of serious conditions including hypertension, raised cholesterol and recently two inpatients have been diagnosed with diabetes..." (Practitioner 6)

Not all the feedback from participants was positive. For some changing practice has been a slow, challenging process...

"Implementation has been quite slow and I think it is due to changing or trying to change colleague's perception to take on something different..." (Practitioner 5)

### Feedback from patients

WSP seems to be highly regarded by patients. Many of the participants that we interviewed described how keen patients were to attend the WSP.

"...in fact group [members] have caught me in the week and reminded me about this week's session." (Practitioner 2)

### Working holistically to promote recovery

A number of participants talked about being more able to meet the holistic needs of the patient.

"WSP provides me with an excellent platform from which to achieve this objective [holistic working]." (Practitioner 1)

"I feel that it [WSP] has got a lot of people looking more holistically at their clients and not making presumptions that they are having their physical needs met by their GP." (Practitioner 3)

"I hope that many more will continue to develop their knowledge and skills, as this will have holistic and therapeutic impact on individuals in our care..."

### Modification of program

There was evidence that practitioners adapted the WSP to fit with their services."...due to the client group, and the high turnover of patients, I have had to modify [*the program*]..." (Practitioner 3)."...because of the client group I needed to be vigilant and seize the moment..." (Practitioner 6)."This was an idea suggested by one of our service users who had used this technique [wake up and shake up] within a school setting".

## Discussion

The WSP may have potential in enhancing the physical health of patients with SMI. The service evaluation published by Smith *et al. *[21] was encouraging; authors reported that 80% of patients completed the two-year program; offered as an adjunct to usual care. When we made the decision to adopt a number pragmatic decisions were taken about implementation that meant that our program would differ in a number of important respects to the original model described by Ohlsen et al [20]. This real-world service evaluation therefore primarily sought to demonstrate that practitioners would engage and retain patients in the program.

As a pilot the total number of patients enrolled on the program was encouraging, however represents only a modest proportion of the SMI population we serve. We recognize that to have a meaningful impact on the physical health of the population that use our services many more practitioners will need to be trained. That said, we have, for the first time, demonstrated that mental health practitioners in a real world setting can and will do physical health checking with SMI patients. The patients enrolled, in terms of demographic characteristics, were representative of SMI patients in the UK. It was disappointing that so few patients completed the WSP; an observation that contrasts with the very high completion rate reported by Smith *et al. *[21]. Understanding this difference is vital in helping managers and practitioners ensure WSP reaches the target population and that resource is used effectively. One possible explanation might be lack of practitioner motivation. Practitioners need to ensure patients progress through the program; if they do not monitor attendance the patients may be more likely to drop out. If this conclusion were upheld, closer performance management of practitioners may be one way of ensuring enhanced patient retention in the program. However, authors of surveys of mental health practitioner attitudes to the physical health needs of patients with SMI observe that mental health practitioners are both motivated and committed to addressing the physical health needs of this population [14]. The feedback from practitioners interviewed suggested that they were generally very positive about the program.

Some of the qualitative observations seem to suggest that the inherent complexity of the WSP may be a barrier to formally retaining patients. Care should be taken to explain the reasons for the measures, tests and data entry required in the first two steps (generally one or two face-to-face session) of the program, as many of the tests may be perceived as not informative in guiding intervention. Practitioners working in a busy and stressful mental health service will undertake the work that is required by the Trust (which does not include WSP data entry) or where there is clear benefit to the patient. It is argued that, perhaps, practitioners do not work to retain patients because of the inherent complexity of the program, especially during the first two sessions. There may be merit in developing and testing a simpler data collection tool that focuses on clinical utility in the health service allowing intervention to adapt to meet local needs and demand.

What can be beyond doubt is the need for physical health intervention in this population. It is positive that so many practitioners have embraced the program. We observed, in common with many other researchers, that the population of SMI patients is generally overweight or obese [13,23]. It is perhaps surprising that such a high proportion of patients were prescribed atypical antipsychotics, which are associated with weight gain. Perhaps this is due to prescriber confidence in using these medicines. Lifestyle factors seem to be major contributory factor to obesity levels. It was disappointing to observe no significant lifestyle improvements, given that reductions in smoking and alcohol use, and an increase in physical activity were observed by Smith *et al. *[21]. This may be explained by the large number of patients for whom follow-up measures were not completed. It is a possibility that there was a lack of fidelity to the effective elements of well-being in the real world service evaluation. Certainly in order to continue with the program, a dramatic improvement in the data collection practice in the service will be required.

The proportion of patients that completed laboratory testing was low. Abnormal results that required intervention were observed in an important proportion of patients tested. Rates of laboratory testing may have been relatively poor because patients needed to be motivated to attend an additional appointment at a pathology laboratory. Practitioners may have also failed to place appropriate emphasis on communicating the importance of laboratory testing with patients. Qualitative feedback from practitioners emphasized the value of the diet/lifestyle element, but with few references to laboratory testing. The current results contrasted with Smith *et al. *[21] and may suggest the need to promote, as part of the WSP training package, the importance and value of this element.

Weight maintenance, rather than reduction, was observed in patients completing the WSP. This is an observation that, we would argue, is a modest positive finding. Authors of naturalist studies of antipsychotic use report weight increases of up to 3 kilograms per year in patients not receiving intervention; maintenance of weight as an outcome can therefore be considered helpful [19]. Of course, weight loss and a consequent reduction in BMI have to be the goal of an SMI well-being program. Weight management programs with more intensive input seem to produce a greater effect, suggesting that the WSP needs to intensified; with increased face-to-face sessions with practitioners [19].

The significant association in patient's blood pressure was intriguing, with approximately equal numbers of patient's moving from being hypertensive to normal and vice versa. It was observed that BMI and BP were correlated, although on closer investigation the increase in BMI categories was not associated with patients becoming hypertensive. Change in blood pressure may be explained by lifestyle factors such as diet or exercise but it was not possible to elicit any evidence of these changing in the sample. What is clear is that a third of the sample had hypertension, and regular monitoring of this in patients with SMI is important.

Although lifestyle risk factors for cardiovascular disease were common in the sample, rates are lower than might be anticipated in a population of patients with SMI. For example, authors conducting epidemiological research have observed that rates of smoking, substance use and alcohol consumption are very high in this population. Additionally, diet tends to be very poor and few patients take regular exercise [5,7,24]. It seems likely that patients are either deliberately or accidentally providing inaccurate information about lifestyle behaviors. This is perhaps surprising given that patients had an existing relationship with practitioners. This is a population with extremely poor self esteem, therefore, discussing, and potentially being criticized for lifestyle behaviors may be threatening to patients. Consequently, these patients may deny engaging in these behaviors as a defense. This apparent discrepancy may emphasize the need for mental health practitioners to be interpersonally skilled at engaging and eliciting information about their patients' physical health.

The WSP focuses almost exclusively on cardiovascular risk factors, and it is important to point out the limitations of this approach. Whilst CVD is the major cause of premature death in SMI patients, increased rates of HIV and cancer have also been observed in this population. For example, Gray *et al. *[25] reviewed the literature on this topic, and demonstrated rates of HIV infection were nine-times greater than in the general population; an observation that may be explained by other lifestyle factors such as unprotected sex. On a more practical, yet important note, high rates of dental caries and poor eye health have been observed in the population [26-29]. If we are to be holistic in our practice; and promote social inclusion as part of our work, these health issues are important to consider. From the qualitative interviews, practitioners recognized the need to adapt and expand the program focusing on a much more diverse range of physical health issues. The Well-Being Support Program must comprehensively address physical health risks that go beyond CVD.

### Limitations of the study

The limitations of the study are similar to those reported by Smith *et al. *[13,21]. The current researchers have no information on patients who did not want to participate in the program, considerable data is missing, and the fidelity of practitioners to the WSP cannot be guaranteed.

## Conclusions

Naturalistic observations were reported from the WSP delivered by mental health practitioners in a secondary care setting. These observations afford considerable learnings for those who work in mental health services and have responsibility for addressing the physical health needs of this population. Mental health workers have to consider physical health to be a serious risk requiring effort and attention.

## Competing interests

Donna Eldridge has no competing interests to disclose, financial or otherwise.

Nicky Dawber has no competing interests to disclose, financial or otherwise.

Richard Gray has received consultancy payments from AstraZeneca, Bristol-Myers Squibb, Cambridge Laboratories, Jannsen-Cilag, Eli Lilly and Company, Otsuka Pharmceutical Europe Ltd. and Pfizer. He has received honorarium payments from all of the above, plus Wyeth.

## Authors' contributions

DE conceived the study. DE and ND designed and carried out the service evaluation and contributed to data analysis. RG analyzed the data and drafted the manuscript. All authors read and approved the final manuscript.

## Pre-publication history

The pre-publication history for this paper can be accessed here:

http://www.biomedcentral.com/1471-244X/11/46/prepub
